# Reply to: Population genetic considerations regarding the interpretation of within-patient SARS-CoV-2 polymorphism data

**DOI:** 10.1038/s41467-024-46262-3

**Published:** 2024-04-16

**Authors:** Chase W. Nelson, Leo L. M. Poon, Haogao Gu

**Affiliations:** 1 Division of Cancer Epidemiology and Genetics, National Cancer Institute, National Institutes of Health, Rockville, MD 20850 USA; 2 Institute for Comparative Genomics, American Museum of Natural History, New York, NY 10024 USA; 3 School of Public Health, LKS Faculty of Medicine, The University of Hong Kong, Hong Kong SAR, China; 4 Centre for Immunology & Infection, Hong Kong Science and Technology Park, Hong Kong SAR, China; 5 HKU- Pasteur Research Pole, School of Public Health, LKS Faculty of Medicine, The University of Hong Kong, Hong Kong SAR, China

**Keywords:** SARS-CoV-2, Evolutionary genetics

**replying to** V. Soni et al. *Nature Communications* 10.1038/s41467-024-46261-4 (2024)

In comments on our paper “Within-host genetic diversity of SARS-CoV-2 lineages in unvaccinated and vaccinated individuals,” Soni et al. argue that the methods we employed for detecting natural selection are unreliable. Our study examined nucleotide diversity (*π*)^1^, the mean number of pairwise differences per nucleotide site, which is a common metric for quantifying within-host viral polymorphism^2^. Comparison of *π* at nonsynonymous (*π*_N_) and synonymous (*π*_S_) sites is thought to provide evidence for positive (*π*_N_ > *π*_S_ or *π*_N_/*π*_S_ > 1) or purifying (*π*_N_ < *π*_S_ or *π*_N_/*π*_S_ < 1) selection acting on amino acid changes^3,4^. This method has been used to study the intrahost evolution of viruses like influenza, often with evidence of positive selection in regions encoding immune epitopes^5^. Intrahost *π*_N_ and *π*_S_ have also been examined in SARS-CoV-2^6–10^, and our study^11^ compared *π*_N_ – *π*_S_ across distinct COVID-19 patient subsets. We found that breakthrough infections in 2- or 3-dose Comirnaty and CoronaVac vaccinated individuals do not show elevated viral *π*_N_ and may not change the direction of selection. These negative conclusions inherently control for viral demographic factors like bottlenecks that operate similarly in each patient, allowing straightforward interpretation of *π*_N_ – *π*_S_ differences.

Soni et al.^12^ challenge our null hypothesis of *π*_N_ – *π*_S_ = 0 (i.e., *π*_N_ = *π*_S_), instead proposing that simulation is necessary for defining a precise expectation under neutrality. Indeed, *π*_N_ – *π*_S_ has widely recognized limitations^13^; for detecting positive selection, it is both overly conservative (may fail to detect positive selection when it has occurred) and susceptible to false positives (may spuriously detect positive selection when it has not occurred). Value is therefore placed on complementing the metric with other approaches. While recognizing these points, we believe the criticisms of Soni et al. may not be entirely valid. In fact, their own simulations demonstrate that selection is often readily detectable using a simple *π*_N_ versus *π*_S_ method.

First, Soni et al. employ analytical methods that do not reflect our study^11^. In our approach, the codon is treated as the observational unit, such that *π*_N_ and *π*_S_ values for each codon are averaged across all 2,820 intrahost samples or subsets thereof. Selection is then evaluated with a Z-test of the null hypothesis *π*_N_ – *π*_S_ = 0 by bootstrapping codons. This detects codon-specific patterns that are consistent across samples; takes advantage of the independent diversity generated in each sample; and compensates for the typically small number of intrahost single nucleotide variants (iSNVs) that pass quality control for any one sample. In contrast, Soni et al.^12^ use the sample as the observational unit and report values of *π*_N_ and *π*_S_ for 200 replicates, analogous to only 200 samples. Their simulations also fail to recapitulate key aspects of the observed biological data, including *π*_N_ – *π*_S_ values and numbers of iSNVs per sample (Supplementary Fig. 1).

Next, Soni et al. report no statistical tests. However, based on data simulated with SLiM^14^, they suggest that large variances make *π*_N_ > *π*_S_ probable even under purifying selection alone. This claim relies on the visual inspection of standard deviations in their Figs. 1–3. To assess it, we used the models of Soni et al. to simulate intrahost data for 100 samples, estimating standard errors of mean *π*_N_ and *π*_S_ as in our study. Purifying selection is highly significant for all models (*P* ≤ 5.0 × 10^−7^, Z-tests) (Supplementary Fig. 1). Purifying selection is detected even using their own sample-based approach (*P* ≤ 1.6 × 10^−6^, Wilcoxon Signed Rank tests). Thus, in contrast to their conclusions, a relatively small number of samples has sufficient statistical power to detect widespread selection using both methods.

Soni et al. then offer several simulations of positive selection. First, directional selection is modelled by introducing a single highly beneficial mutation (i.e., a selective sweep) in the context of a neutral/deleterious distribution of mutational fitness effects (DFE). Because the fraction of nonsynonymous mutations that are beneficial (*f*_b_) in this scenario is ~0.00007%, it is not surprising that *π*_N_ – *π*_S_ fails to detect positive selection. Specifically, *π*_N_ – *π*_S_ is tailored to detecting pervasive (multi-site), incomplete positive selection that is ‘caught in the act’. Population genetics theory suggests that the substitution of beneficial mutations takes an average of approximately $2{{{{\mathrm{ln}}}}}(2{N}_{e}s)/s$ generations^15^. For selection coefficients (*s*) of 0.01–0.1 and intrahost effective population sizes (*N*_e_) of 10^3^–10^5^, this implies an average of 45–644 days for SARS-CoV-2 (i.e., 106–1,520 replication cycles of 610 minutes^16^). A selective sweep is therefore not expected to complete over the course of a typical acute infection within a host. Furthermore, within-host viral evolution is likely to involve trade-offs, compensatory mutations, shifting fitness landscapes, and potentially balancing selection as a result of intrahost heterogeneity and frequency dependence^17^. In all cases, segregating nonsynonymous mutations will elevate *π*_N_.

In a second scenario of positive selection, Soni et al. set *f*_b_ to 1.0% or 9.7% (*s* = 0.05–0.13) in the context of a DFE derived from Flynn et al. for Mpro (nsp5)^18^. We again used their models to simulate 100 samples (Fig. 1). Although they claim that *π*_N_ – *π*_S_ cannot detect selection, positive selection was highly significant at the whole-genome level for *f*_b_ = 9.7% (*π*_N_/*π*_S_ = 4.43, *P* < 2.2 × 10^−16^), whereas purifying selection was detected for *f*_b_ = 1.0% (*π*_N_/*π*_S_ = 0.90, *P* = 0.0033; Z-tests). Thus, under the simulation parameters of Soni et al., positive selection becomes highly significant for *f*_b_ somewhere in the range 1–10%, due to multiple beneficial mutations segregating at intermediate frequencies.

**Fig. 1:**
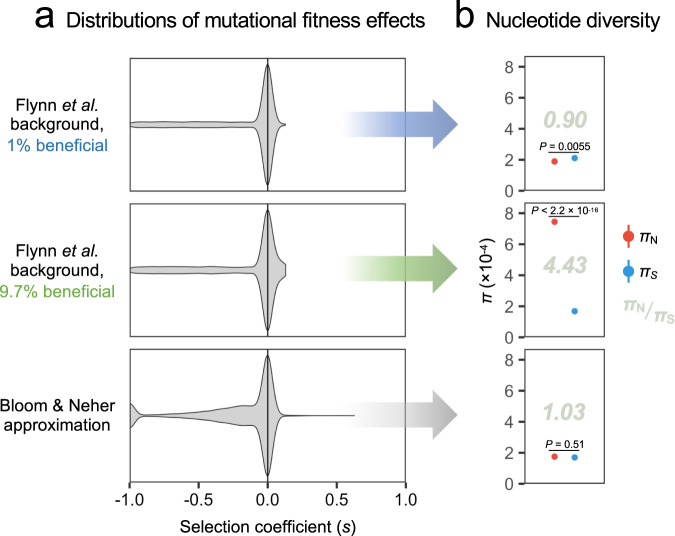
Characterization of simulated data generated using models that allow multiple beneficial mutations. The SLiM^14^ simulations of Soni et al.^12^. were modified to generate 100 whole-genome (30 kbp) samples for each of three distributions of mutational fitness effects (DFEs) based on Flynn et al.^18^ and Bloom & Neher^19^. Flynn et al.^18^ refers to a DFE background estimated for Mpro (nsp5), with either 1.0% (blue text and arrow) or 9.7% (green text and arrow) of mutations beneficial (selection coefficients [*s*] = 0.05–0.13). Bloom & Neher^19^ (grey arrow) refers to a DFE estimated from publicly available viral consensus sequence data, where the fractions of each mutation effect type were set to the whole-genome values given in Table 1 (bottom row). For the latter, *s* values were approximated by dividing fitness effects (range −7.14–6.17) by 7.14 (maximum absolute value), yielding a range of −1.0–0.86. These values were simulated as lethal = −1.0; deleterious = gamma (mean −0.32, shape 1.70); neutral = 0.0; and beneficial = exponential (mean 0.087). For the gamma distribution shape parameter, a maximum likelihood estimate was obtained from the absolute values of all negative *s* using the MASS::fitdistr() function in R. All other parameters were retained from the scripts of Soni et al.: mutation rate = 2.135 × 10^−6^ per site per cycle; recombination rate = 5.5 × 10^−5^ per site per cycle; infection bottleneck size = 1; carrying capacity = 100,000; runtime = 168 cycles (https://github.com/vivaksoni/Gu_etal_2023_response, accessed 2023/09/26). Simulated data were analyzed using the method of our original study^11^, i.e., eliminating iSNVs with frequency <2.5% and estimating *π*_N_ – *π*_S_ with codon-based bootstrapping. **a** DFEs for nonsynonymous mutations. Violin plots show the emergent *s* distributions of the three DFE models, each determined by simulating 10,000 mutations. **b** Nucleotide diversity under each DFE. Error bars show standard errors of mean *π*_N_ (red) and *π*_S_ (blue), each determined using 1,000 bootstrap replicates (codon unit, with codon values calculated as means across all 100 samples). *P* values refer to two-sided Z-tests of *π*_N_ = *π*_S_ (three tests; no adjustment for multiple tests). *π*_N_/*π*_S_ ratios are displayed in grey text; for comparison, the mean empirical *π*_N_/*π*_S_ value observed across all biological samples in our original study^11^ was 0.62. Scripts, analysis code, input data, and intermediate files are available at 10.5281/zenodo.10552831. Source data are provided as a Source Data file.

To estimate *f*_b_ for SARS-CoV-2, we utilized the fitness effect calculations of Bloom and Neher^19^. The central 95% of synonymous mutational effects was considered a null (neutral) distribution, such that nonsynonymous mutations were classified as beneficial if their effects fell above the 97.5^th^ percentile of synonymous mutations. Results are summarized in Table 1. For the whole genome, *f*_b_ is 1.5%. For individual ORFs, *f*_b_ ranges from 0.8% (ORF1ab) to 6.6% (ORF7a). For sliding windows of 30 codons such as used in our study^11^, *f*_b_ ranges from 0% to 13.7%. Maximum regional *f*_b_ values occur near Spike codons ~127–175 and ~461–512, overlapping the antigenically important amino-terminal (NTD) and receptor-binding (RBD) domains^20^. Thus, at the levels of whole ORFs and functional domains, *f*_b_ for SARS-CoV-2 often falls in a range that allows detection of positive selection by *π*_N_ – *π*_S_.

**Table 1:** Estimated fractions of SARS-CoV-2 nonsynonymous mutations that are lethal, deleterious, neutral, and beneficial

Region	*n*	Lethal	Deleterious	Neutral	Beneficial (*f*_b_)
ORF1ab	41757	10.0% (9.7–10.3%)	46.0% (45.6–46.5%)	43.2% (42.7–43.7%)	0.8% (0.7–0.9%)
S	7797	8.8% (8.2–9.5%)	39.2% (38.1–40.3%)	48.9% (47.8–50.0%)	3.1% (2.7–3.5%)
ORF3a	1640	1.2% (0.8–1.9%)	18.7% (16.8–20.6%)	76.2% (74.1–78.2%)	3.9% (3.0–5.0%)
E	448	6.0% (4.1–8.8%)	52.5% (47.7–57.1%)	39.5% (35.0–44.2%)	2.0% (1.0–3.9%)
M	1317	12.0% (10.3–13.9%)	46.7% (44.0–49.4%)	40.3% (37.7–43.0%)	1.0% (0.5–1.7%)
ORF6	374	0.3% (0.0–1.7%)	15.2% (11.8–19.4%)	81.6% (77.2–85.3%)	2.9% (1.6–5.4%)
ORF7a	715	0% (0.0–0.7%)	7.1% (5.4–9.3%)	86.3% (83.5–88.7%)	6.6% (4.9–8.7%)
ORF7b	251	0% (0.0–1.9%)	9.2% (6.0–13.6%)	87.3% (82.3–91.0%)	3.6% (1.8–6.9%)
ORF8	712	0% (0.0–0.7%)	12.6% (10.3–15.4%)	82.3% (79.3–85.0%)	5.1% (3.6–7.0%)
N	2502	7.7% (6.7–8.8%)	26.1% (24.4–27.9%)	62.8% (60.9–64.7%)	3.4% (2.7–4.2%)
ORF9b	585	6.0% (4.3–8.3%)	26.3% (22.8–30.1%)	62.1% (58.0–66.0%)	5.6% (4.0–7.9%)
ORF10	226	1.3% (0.3–4.1%)	23.9% (18.6–30.1%)	73.5% (67.1–79.0%)	1.3% (0.3–4.1%)
S:135–164	182	0% (0.0–2.6%)	16.5% (11.6–22.9%)	69.8% (62.5–76.2%)	13.7% (9.3–19.8%)
S:465–494	219	7.3% (4.4–11.8%)	25.6% (20.0–32.0%)	55.3% (48.4–61.9%)	11.9% (8.0–17.1%)
**Genome**	**58324**	**9.1% (8.8–9.3%)**	**42.0% (41.6–42.4%)**	**47.4% (47.0–47.8%)**	**1.5% (1.4–1.6%)**

Results are based on the fitness effect estimates of Bloom and Neher^19^, downloaded from https://github.com/jbloomlab/SARS2-mut-fitness (aamut_fitness_all.csv, public_2023-10-01 dataset; accessed 2023/10/05). The central 95% of synonymous mutational fitness effects were used as the null (neutral) distribution. Specifically, nonsynonymous mutations were classified by their fitness effects as (1) lethal if ≤ −3.95; (2) deleterious if > −3.95 and < −1.75; (3) neutral if ≥ −1.75 and <1.20; and (4) beneficial if ≥ 1.20 (−3.95 is the median effect of stop mutations and −1.75–1.20 is the central 95% of synonymous mutational effects). Ranges indicate 95% binomial confidence intervals. Sites in putative overlapping ORFs^25^ were included. S:135–164 and S:465–494 refer to the 30-codon windows with the highest values of *f*_b_. The final row provides results for the full coding genome (bold). Scripts, analysis code, input data, and intermediate files are available at 10.5281/zenodo.10552831.

Last, we modified the simulations of Soni et al. by introducing a DFE based on the nonsynonymous fitness effect estimates of Bloom and Neher^19^. Whole-genome mutation effect fractions (bottom row of Table 1) were used as a background. Deleterious and beneficial selection coefficients (*s*) were modelled using gamma (mean = −0.32, shape = 1.70) and exponential (mean = 0.087) distributions, respectively. Under these parameters, at the whole-genome level, selection was not significant (*π*_N_/*π*_S_ = 1.03, *P* = 0.51) (Fig. 1b bottom). At the level of 30-codon sliding windows, we considered regions with *π*_N_ > *π*_S_ to be candidates for positive selection at various *P* value cut-offs, detecting 131 true positives (windows with at least one beneficial mutation) and 0 false positives for *P* < 0.0124. Thus, even under a nonideal scenario where the precise genomic targets of selection (codons with beneficial mutations) differ stochastically across samples, sliding windows are a reasonable candidate generator for regions undergoing positive selection.

All simulation results reported by Soni et al. and herein are subject to many limitations and likely do not reflect biological reality. First, DFEs were derived from functional assays^18^ or clinical isolates^19^ and therefore describe between-host evolution, but it is known that purifying selection is weaker within hosts^6,21^. Second, the models may contain important misspecifications, including (1) sequencing coverage of only 100 effective reads (median coverage in our study was 20,782 reads); (2) 2/3 of sites nonsynonymous (compared to ~3/4 in most real ORFs); (3) *s* > 1.0 in a SLiM non-Wright-Fisher context (Soni et al. Figure 2); (4) intrahost dynamics that may deviate from expected viral population sizes; and (5) no tendency for the same site to be under similar selection pressures across multiple samples (e.g., no convergent selected changes). Model complexity potentiates increased misspecification bias, and it is important for both biological parameters and analytical methods to match between simulated and empirical data.

To summarize, *π*_N_ – *π*_S_ has limitations. Care must be exercised, as factors other than positive selection can yield *π*_N_ > *π*_S_, especially in short genome regions where *π*_S_ is subject to stochastic fluctuation. The expected value of *π*_N_/*π*_S_ depends on *f*_b_ and DFE properties. More work is needed to determine the precise values of *f*_b_ necessary for detecting positive selection, intrahost DFEs, and additional criteria for lowering the false-discovery rate (e.g., a minimum *π*_N_ cutoff). All parameters are likely to vary by host, virus, lineage, and many other contexts. SLiM offers unprecedented opportunities for simulating complex evolutionary scenarios in order to test specific hypotheses^14^. Nevertheless, we maintain that simple methods like *π*_N_ – *π*_S_ have value. In the same way, simple *d*_N_/*d*_S_ analyses continue to yield highly informative results^22^ even though viral consensus sequences do not incorporate real-world complexity, and each site in a genome may in reality follow its own ‘model’ of evolution which changes over time^23^. As the aphorism suggests, the question is not whether models are realistic, but rather whether they are useful^24^. While more advanced methods are always welcome, there is no one ‘right’ way to analyze evolutionary genomics data^23^.

## Reporting summary

Further information on research design is available in the Nature Portfolio Reporting Summary linked to this article.

### Supplementary information

Supplementary Information

Reporting Summary

### Source data

Source Data

## Data Availability

All input data, intermediate files, and simulated data have been deposited at Zenodo under accession code 10.5281/zenodo.10552831. Data for estimating *f*_b_ were obtained from the aamut_fitness_all.csv file of Bloom and Neher^19^ (public_2023-10-01 dataset; accessed 2023/10/05). Figure source data are provided as a Source Data file. Source data are provided with this paper.

## Code availability

Simulation and analysis scripts have been deposited at Zenodo under accession code 10.5281/zenodo.10552831.
