# Supplementary material for: Reply to: Population genetic considerations regarding the interpretation of within-patient SARS-CoV-2 polymorphism data
Source: Nat Commun. 2024 Apr 16;15:3239. doi: 10.1038/s41467-024-46262-3 (PMC11021549; doi:10.1038/s41467-024-46262-3)
Supplement: Supplementary file 1 — Supplementary Information [file 41467_2024_46262_MOESM1_ESM.pdf]

Supplementary Information

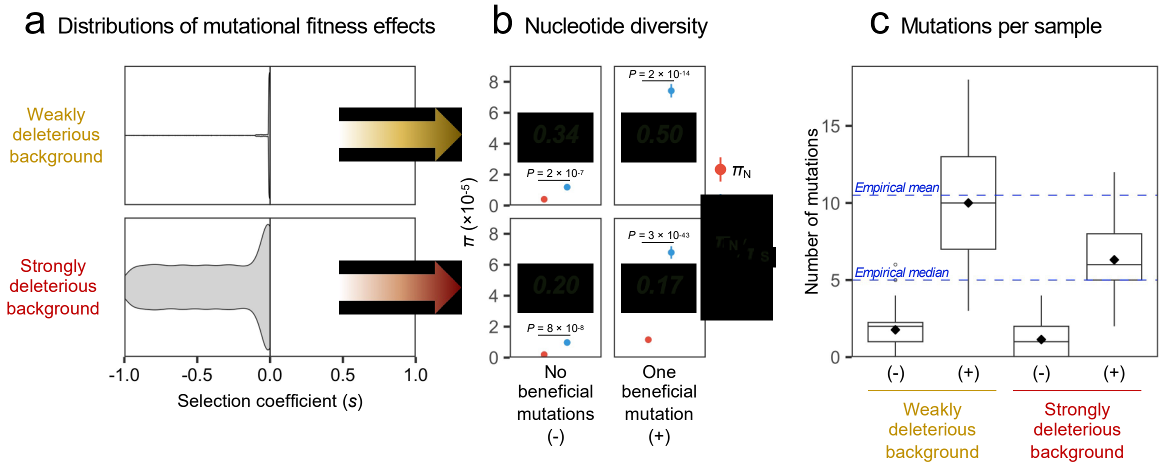

Supplementary Figure 1. Characterisation of simulated data generated using the models of Soni *et al.* The SLiM[14] simulations of Soni *et al.*[12] were used to generate 100 whole-genome (30 kbp) samples for each of four parameter combinations: weakly (yellow text and arrow) vs. strongly (red text and arrow) deleterious mutation backgrounds, and zero (-) vs. one (+) beneficial mutation introduced. Simulated data were analysed using the method of our original study[11], i.e., eliminating iSNVs with frequency <2.5% and estimating $\pi_N - \pi_S$ using a codon-based method. All original simulation parameters of Soni *et al.* were used, and models with one beneficial mutation (+) were analysed at fixation (complete selective sweep). **a** Distribution of mutational fitness effects (DFE) for nonsynonymous mutations. Violin plots show the emergent selection coefficient distributions of the two DFE background models, each determined by simulating 10,000 mutations. Fitness is absolute in SLiM non-Wright-Fisher models, so selection coefficients (*s*) with values >1 do not have a straightforward interpretation. **b** Nucleotide diversity under each of the four parameter combinations. Error bars show standard errors of mean π_N (red) and π_S (blue), each determined using 1,000 bootstrap replicates (codon unit, with codon values calculated as means across all 100 samples). *P* values refer to two-sided Z-tests of $\pi_N = \pi_S$ (four tests; no adjustment for multiple tests). π_N/π_S ratios are displayed in grey text. For comparison, the mean empirical π_N/π_S value observed across all biological samples in our original study[11] was 0.62. **c** Mutations per sample. Boxplots show distributions of the number of mutations per simulated sample, where black diamonds denote means. The horizontal line inside the box shows the median, upper and lower edges of the box indicate the first and the third quartiles, and whiskers extend to 1.5 interquartile range from the edges. For comparison, blue dashed lines show the empirical mean (10.5 iSNVs per sample; top) and median (5 iSNVs per sample; bottom) values observed across all biological samples in our original study[11]. Simulation scripts were downloaded from https://github.com/vivaksoni/Gu_etal_2023_response (accessed 2023/06/16), and additional code was added to obtain samples of 100 individual genomes (analogous to effective sequencing reads). Scripts, analysis code, input data, and intermediate files are available at https://zenodo.org/doi/10.5281/zenodo.10552831. Source data are provided as a Source Data file.
